# Prevalence and distribution of shoveling and double shoveling non-metric traits in Mysuru population: A cross sectional study

**DOI:** 10.1016/j.jobcr.2025.12.006

**Published:** 2025-12-10

**Authors:** G.R. Aiswarya, H.S. Sreeshyla, Swathi Kumareswar

**Affiliations:** a Former PG-MSc Forensic Odontology, JSS Dental College and Hospital, JSSAHER, Mysuru, India; b Department of Oral Pathology and Microbiology, JSS Dental College and Hospital, JSSAHER, Mysuru, India; c Western Sydney University, Australia

**Keywords:** Shoveling, Non-metric traits, Forensic, Anthropology, Personal identification, ASUDAS, Dimorphism

## Abstract

**Background:**

Non-metric dental traits play a pivotal role in anthropological and forensic investigations. Shovel-shaped incisors, in particular, serve as valuable indicators of population affinity and individual identification. This study aimed to assess the frequency and grading of the non-metric shoveling and double shoveling traits in the Mysuru population using the Arizona State University Dental Anthropology System (ASUDAS) scoring criteria.

**Methodology:**

A cross-sectional descriptive study was conducted in Mysuru, among 382 individuals (190 males and 192 females) aged 18–40 years. Traits were assessed clinically using ASUDAS plaques. Data were recorded and analyzed using descriptive statistics and chi-square tests to determine prevalence and statistical significance across gender, arch, quadrant and tooth type**.**

**Results:**

Shoveling was highly prevalent in the population (95.5 %), especially in upper central incisors (92.2 %), followed by upper lateral incisors (69.3 %). In contrast, lower anterior teeth exhibited minimal expression (<3 %). Double shoveling was rare, observed in only 1.0 % of individuals. No statistically significant difference in trait prevalence was found between genders or sides (p > 0.05). However, shoveling was significantly more common in the upper arch than the lower (p = 0.000). Grading analysis revealed Grade 1 shoveling as the most common form, particularly in upper central and lateral incisors. Double shoveling was mostly absent, with only trace levels (Grade 1) being noted.

**Conclusion:**

The study highlights the prevalence of shoveling, especially in the maxillary anterior teeth among the Mysuru population. The results support the forensic and anthropological value of these traits in personal identification and population studies.

## 1 Introduction

Forensic anthropology and forensic odontology, although distinct, frequently collaborate closely in forensic investigations. This interdisciplinary collaboration enhances the accuracy and comprehensiveness of forensic analyses, ultimately contributing to the resolution of criminal cases and justice.^1^^,^^2^ The distinctive and individualistic traits provide valuable information for personal identification, ancestry determination, bite mark analysis, and the identification of victims in mass disasters.^1^

Non-metric dental traits are minor variations in tooth shape and structure that are not measured on a continuous scale.^3^ These encompass characteristics such as the presence or absence of specific cusps, grooves, or ridges on the crowns or roots of teeth, as well as variations in tooth morphology and size.^4^ These traits, which are often unique to each person, prove invaluable in matching dental records with those of an unidentified body or a suspect and can also provide valuable information about the population to which an individual belongs.^5^^,^^6^

Shovel shaped incisors form one of the significant non-metric dental traits. They are considered an important parameter observed in human dentition which can aid in predicting the main ethnic difference between populations in anthropometric studies.^7^ This trait is seen with high frequency among Mongoloids and has a relatively smaller incidence among Caucasoids.^7^, ^8^, ^9^, ^10^ Shoveling is considered as a polygenic inherited trait, that exhibits in some modern human populations.^10^, ^11^, ^12^ The scaling of this feature was first proposed by Hardlick (1920), plaque was developed by Dahlberg (1956), and an expanded classification was developed by Scott (1973).^13^

The hallmark of shoveling is pronounced lingual marginal ridges. A related trait on the labial surface is referred to as double-shoveling.^14^ Generally, labial marginal ridges are never as pronounced as lingual marginal ridges, though the differences between grades can be subtle.^4^ When the double-shoveling is present, it is typically more pronounced on the mesial than the distal margin.^4^ This trait is observed on Upper central Incisors.^4^^,^^15^ The standard reference was developed by Dahlberg in 1956 and ASU procedure by Turner and Laider Dowda in 1979.^15^

The Arizona State University Dental Anthropology System (ASUDAS) has scoring and analysis for each dental nonmetric trait with a standard protocol based on strict definitions and three-dimensional dental plaques.^4^ However, even with strict features, visual references and the observer's experience used to control visual scoring, subjectivity persists.^4^ The traits observed by the ASUDAS are apparent, reliable and can be identified even if the dentition has become degraded.

Studies conducted on the shoveling trait within India and internationally, have shown the importance of this non-metric dental trait in population differentiation and forensic identification.^8^, ^9^, ^10^, ^11^, ^12^^,^^15^, ^16^, ^17^, ^18^, ^19^, ^20^, ^21^, ^22^, ^23^, ^24^, ^25^, ^26^, ^27^ Pillai et al. reported that non-metric traits show significant differences among different subgroups in Gujarat, with shoveling being the only trait showing significant sexual dimorphism.^20^ Similarly, other studies too have demonstrated the potential of shoveling as valuable diagnostic tools for classifying and characterizing ethnic diversity, in understanding human variation, evolutionary trends, and in forensic identification. The distinct prevalence patterns observed among different studies highlights the importance of regional studies in understanding genetic and environmental influences on dental morphology.^8^, ^9^, ^10^, ^11^, ^12^^,^^15^, ^16^, ^17^, ^18^, ^19^, ^20^, ^21^, ^22^, ^23^, ^24^, ^25^, ^26^, ^27^

Apparently there are not many studies done to record the prevalence and distribution of Shoveling and Double Shoveling Non-Metric Traits regionally. Mysuru city in southern part of India comes under the state of Karnataka. As there are no such studies done in this region, this study was conducted with the following objectives to assess the frequency of non-metric shoveling trait using ASUDAS Scoring criteria.

1. To record the non-metric parameters – shoveling and double shoveling in the Maxillary and Mandibular anterior teeth of Mysuru population
2. To compare these nonmetric parameters of anterior teeth among different genders of Mysuru population.
3. To compare these non-metric parameters between the Maxillary and Mandibular anterior teeth and between the right and left sides.

## 2 Methodology

The study is cross-sectional, descriptive, done for the duration of 6 months. The participants were from Mysuru, a city in Karnataka state in India. Patients and accompanying persons aged 18–40 years visiting the outpatient department of JSS Dental College and Hospital, Mysuru, constituted the study sample. With an absolute precision of 5 % and confidence level of 95 %, a sample size of 382 was determined and random sampling was used. The study was approved by the institutional ethical committee and an Informed consent was obtained from each participant before the beginning of the study.

## 3 Inclusion and exclusion criteria

Participants with completely erupted and non-attrited maxillary and mandibular anterior teeth were considered for the study. Participants with partially erupted maxillary and mandibular anterior teeth, maxillary and mandibular anterior teeth showing regressive alterations, cases with retained deciduous maxillary and mandibular anterior teeth were excluded from the study.

A pilot study was done prior to the actual study among 10 males and 10 females. The presence of the trait along with its grading was recorded in the proforma using clinical examination. The Arizona State University Dental Anthropology System (ASUDAS) scoring system was used for grading the shoveling and Double shoveling among the Incisors and Canines. All the incisors were examined for the presence of shoveling and double shoveling, and all canines were assessed for shoveling alone. The recording was done separately for each incisors. This pilot study helped to standardize the recording of shoveling and Double shoveling. Inter-observer and Intra-observer error in grading the traits was tested with a subsample of 40.

Instruments used for examination and data recording included Intra-oral Mirror, Mouth Mirrors, Probes, ASUDAS Grade sheet and Proforma for Grading the traits. Participants were made to sit comfortably on the dental chair. Recording of the traits were done by examining the oral cavity under bright light conditions. Ambiguous cases were handled by examination of such case by all the investigators and coming into consciences regarding the grading. Once observations are documented on the proforma, the data was transferred to a Microsoft Excel sheet.

Descriptive statistics, including frequencies and percentages, as well as chi-square tests, were employed to analyze the prevalence of anterior non-metric traits in the Mysuru population. All statistical analyses were performed using IBM SPSS Statistics (version 26.0). Descriptive statistics were calculated for the overall prevalence of each trait. Chi-square (χ^2^) tests of independence was used to assess the association between the presence of each dental trait and gender, as well as arch (upper/lower) and side (right/left). The rationale for using this test is that it is the most appropriate for analyzing categorical data to determine if observed distributions differ significantly from those expected by chance. A p-value of less than 0.05 was considered statistically significant.

## 4 Results

The total of 382 participants were studied, with 190 males and 192 females. The findings of the study revealed significant variations in the prevalence of shoveling traits among the Mysuru population.

## 5 Prevalence

Shoveling was very common, with 95.5 % of individuals showing this trait. Double shoveling was found to be very rare, with only 1.0 % of individuals displaying this trait (Table 1).

**Table 1:** Prevalence of Shoveling and Double Shoveling in the study population.

Traits	Frequency	Percentage
Shoveling	365	95.5
Double Shoveling	4	1.0
Total	369	96.6

## 6 Sexual dimorphism

**Fig. 1:**
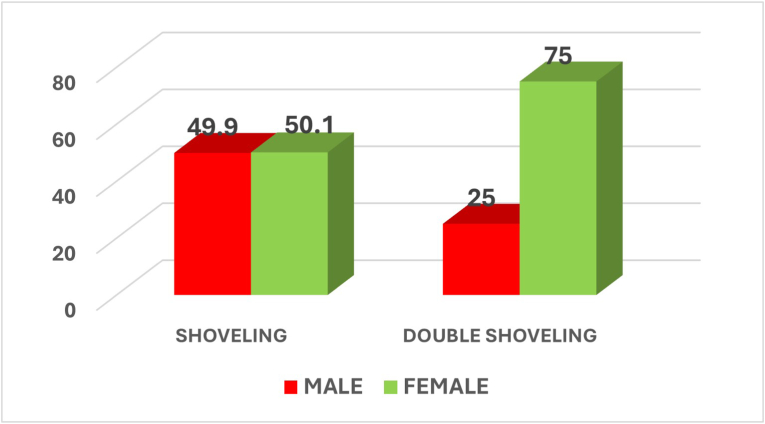
Graph showing the percentage of shoveling and double shoveling among males and females.

On comparison between the genders, shoveling showed an almost equal prevalence among males and females, with a p-value of 0.509 indicating no statistically significant difference. Double shoveling was more common in females (75 %) than males (25 %), and the p-value of 0.315 indicated no statistically significant difference **(**Fig. 1).

## 7 Arch dimorphism

**Fig. 2:**
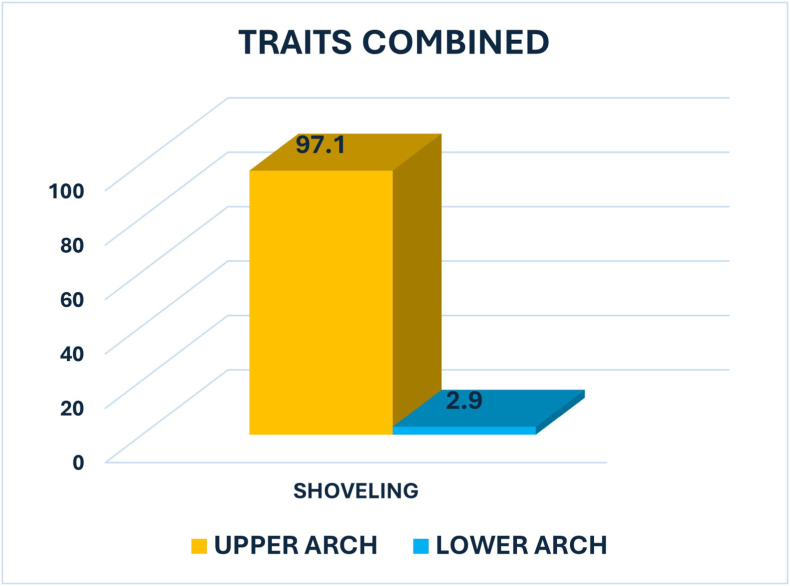
Graph showing the percentage of Shoveling between Upper and Lower Arch.

Shoveling was significantly more common in the upper arch (97.1 %) than in the lower arch (2.9 %), with a p-value of 0.000 indicating a highly significant difference **(**Fig. 2)**.**

## 8 Quadrant dimorphism

**Fig. 3:**
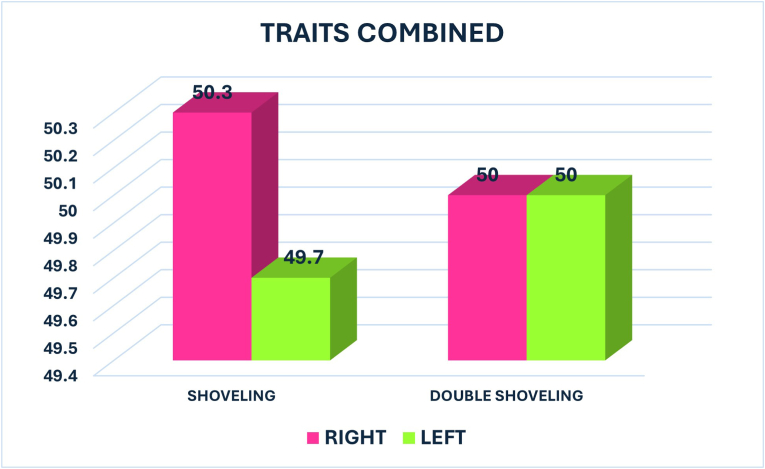
Graph showing the percentage of shoveling and double shoveling between Right and Left side.

Shoveling was nearly equally prevalent between the right (50.3 %) and left (49.7 %) sides, with a p-value of 0.589, also indicating no significant difference. Double shoveling had equal prevalence on both sides (50 %), with a p-value of 0.702, showing no significant difference (Fig. 3).

**Fig. 4:**
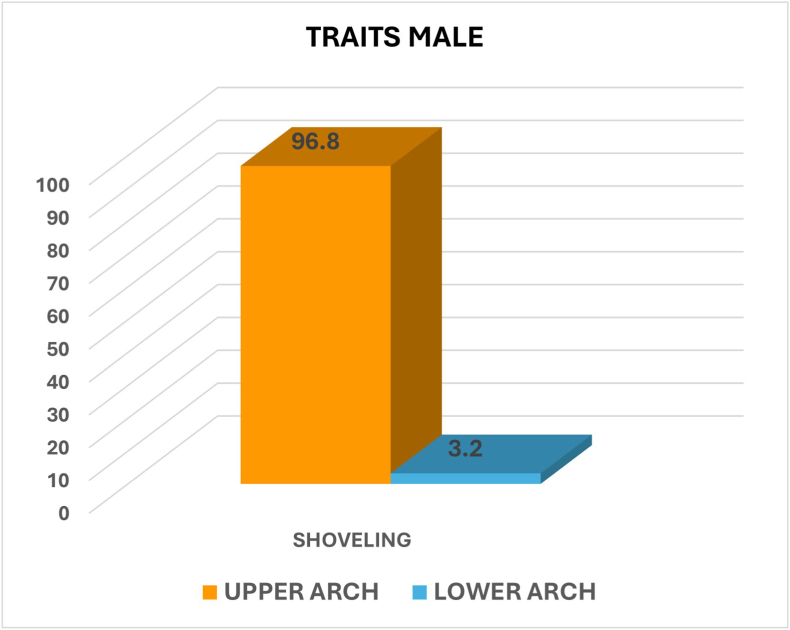
Graph showing the percentage of shoveling between upper and lower arch among males.

**Fig. 5:**
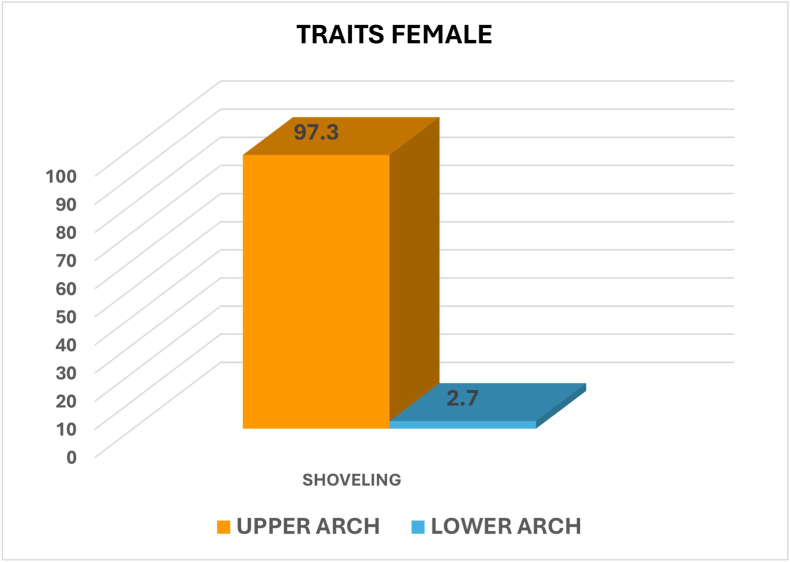
Graph showing the percentage of shoveling between upper and lower arch among females.

Among males, Shoveling was significantly more prevalent in the upper arch (96.8 %) compared to the lower arch (3.2 %), with a highly significant p-value (p = 0.000). Similarly, among Females, shoveling was significantly more prevalent in the upper arch (97.3 %) compared to the lower arch (2.7 %), with a highly significant p-value (p = 0.000) (Fig. 4, Fig. 5).

**Fig. 6:**
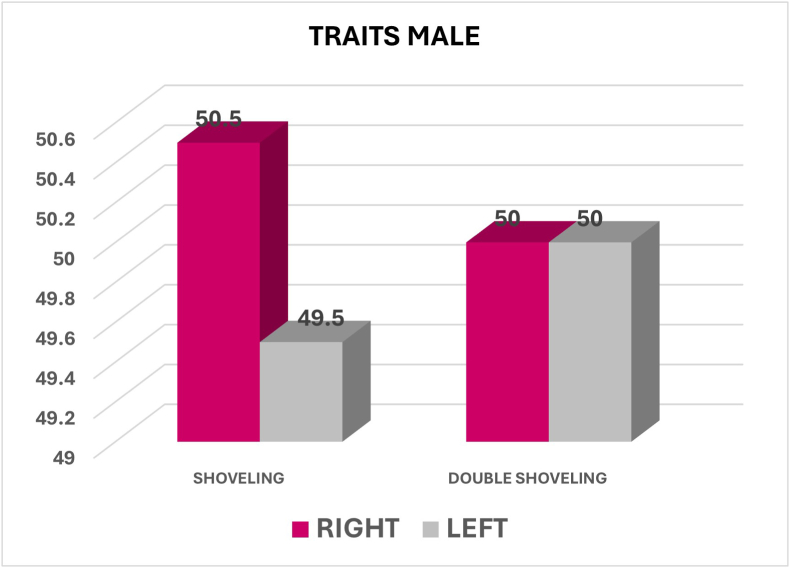
Graph showing shoveling and double shoveling between right and left side among males.

**Fig. 7:**
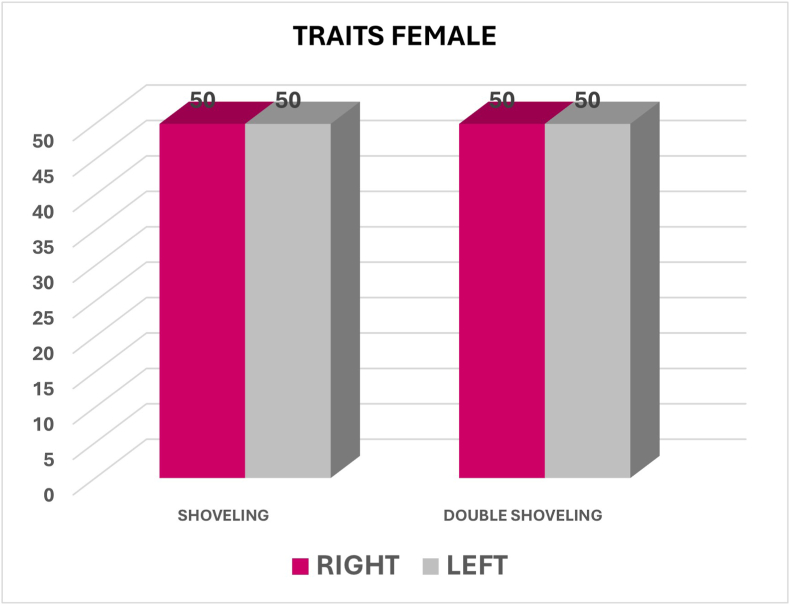
Graph showing shoveling and double shoveling between right and left side among females.

Among males, Shoveling showed almost equal prevalence between the right (50.5 %) and left (49.5 %) sides and was nonsignificant (p = 0.583). Double shoveling also showed equal prevalence on right and left side (50 %), with no significance (p = 0.745). In Female subjects, both Shoveling and Double shoveling showed equal prevalence between right and left side (50 %), with no statistical significance (p = 0.595) (Fig. 6, Fig. 7).

**Fig. 8:**
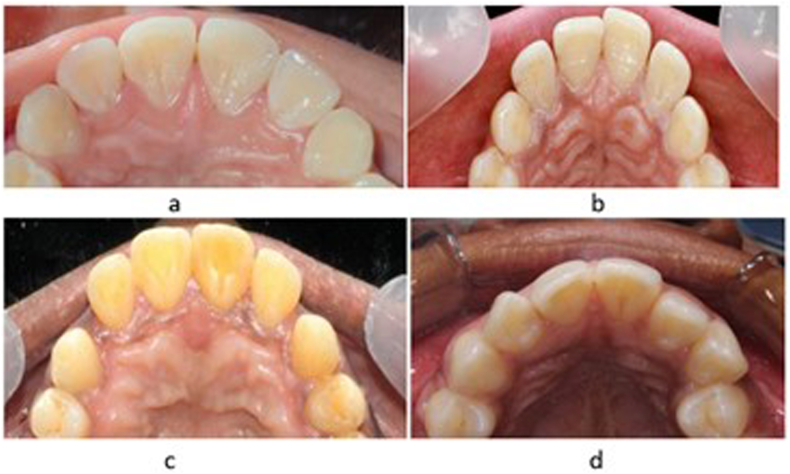
a. Grade 1 shoveling on maxillary central and lateral incisors b. Grade 1 shoveling on maxillary central and grade 2 on lateral incisors c. Grade 3 shoveling on maxillary central incisors and grade 1 on lateral incisors d. Grade 2 shoveling on maxillary central and lateral incisors.

## 9 Class dimorphism

Among the different types of incisors, shoveling was found more in the upper central incisors (92.2 %), followed by the upper lateral incisors 69.3 % and upper canines (20.1 %). In contrast, the lower anterior teeth exhibited significantly lower frequencies, with 2.8 % in the lower central incisors, 2.6 % in the lower lateral incisors, and 2.09 % in the lower canines. This pattern suggests that shoveling is predominantly an upper incisor trait, with minimal expression in the lower dentition.

## 10 Grading in different class of teeth (Fig. 8)

The following types of grading in shoveling and double shoveling were observed across different teeth in our study, highlighting distinct grading patterns. For the upper right central incisors (UCI-R), Grade 1 was the most prevalent at 58.1 %, followed by Grade 2 at 30.6 %, Grade 3 at 4.7 %, and Grade 4 at 0.5 %. Higher grades were less frequent, collectively comprising less than 6 % of the sample. For the upper left central incisors (UCI-L), Grade 1 was the most common, occurring in 58.1 % of cases, with Grade 2 at 30.9 %, Grade 3 at 4.7 %, and Grade 4 at 0.5 %, and Grade 5 at 0.3 %.

In the upper right lateral incisors (ULI-R), Grade 1 was the most prevalent, accounting for 48.2 %, followed by Grade 2 at 14.4 %, Grade 3 at 5.8 %, Grade 4 at 0.5 %, and Grade 5 at 0.5 %. For the upper left lateral incisors (ULI-L), Grade 1 was the most common, occurring in 50.0 % of cases, followed by Grade 2 at 12.8 %, Grade 3 at 5.5 %, Grade 4 at 0.5 %, and Grade 5 at 0.5 %.

In the right upper canines (UC-R), Grade 0 was predominant at 79.1 %, followed by Grade 1 at 18.3 %, Grade 2 at 2.1 %, and Grade 3 at 0.5 %. For the left upper canines (UC-L), Grade 0 was also predominant at 80.6 %, with Grade 1 following at 17.8 %, and Grade 2 at 1.6 %

In the lower central incisors (LCI-R & L), Grade 0 was predominant at 97.1 %, with Grade 1 minimal at 2.9 %. For the lower lateral incisors (LLI-R & L), Grade 0 was observed in 97.4 % of cases. Grade 1 was minimal at 2.4 %, and Grade 3 was rare, at 0.3 %. In the lower canines (LC-R & L), Grade 0 was predominant at 97.9 %, with Grade 1 minimal at 2.1 %.

Our study observed varying degrees of double shoveling across different teeth, highlighting distinct grading patterns. For the upper central incisors on the right (UCI-R), double shoveling was observed with Grade 0 (absence) in 99.0 % of cases. Grade 1 (trace) was the most prevalent, occurring in 1 % of cases. Similarly, for the upper central incisors on the left (UCI-L), Grade 0 (absence) was observed in 99.0 % of cases, Grade 1 present in 1.0 % of cases. In the upper right and left lateral incisors (ULI-R), Grade 1 was observed in 0.3 % of cases (Table 2).

**Table 2:** Grading of Shoveling and Double Shoveling in the study population.

Trait	Grades							
	0	1	2	3	4	5	6	7
Shoveling UCI-R	22(5.8 %)	222(58.1 %)	117(30.6)	18(4.7 %)	2(0.5 %)	1(0.3 %)	–	–
Shoveling UCI-L	21(5.5 %)	222(58.1 %)	118(30.9 %)	2(4.7 %)	2(0.5 %)	1(0.3 %)	–	–
Shoveling ULI-R	117(30.6 %)	184(48.2 %)	55(14.4 %)	22(5.8 %)	2(0.5 %)	2(0.5 %)	–	–
Shoveling ULI-L	117(30.6 %)	191(50 %)	49(12.8 %)	21(5.5 %)	2(0.5 %)	2(0.5 %)	–	–
Shoveling UC-R	302(79.1 %)	70(18.3 %)	8(2.1 %)	2(0.5 %)	–	–	–	–
Shoveling UC-L	308(80.6 %)	68(17.8 %)	6(1.6 %)	–	–	–	–	–
Shoveling LCI-R &L	371(97.1 %)	11(2.9 %)	–	–	–	–	–	–
Shoveling LLI-R&L	372(97.4 %)	9(2.4 %)	–	1(0.3 %)	–	–	–	–
Shoveling LC-R &L	374(97.4 %)	8(2.1 %)	–	–	–	–	–	–
Double shoveling UCI-R&L	378(99 %)	4(1.0 %)	–	–	–	–	–	–
Double shoveling ULI-R&L	381(99.7 %)	1(0.3 %)	–	–	–	–	–	–

## 11 Measure of agreement

The Measure of Agreement of the non-metric dental traits in the study demonstrated perfect kappa values (κ = 1.000), indicating complete intra-observer agreement for Shoveling and Double shoveling. This high level of agreement for the computed traits underscores the reliability and consistency of the examination methodology employed in this study (Table 3).

**Table 3:** Intra-observer reliability in the study population.

Measure of Agreement	Kappa Value
Shoveling	1.000
Double shoveling (UCI)	1.000
Double shoveling (ULI)	a

a No statistics are computed.

## 12 Discussion

The morphology subfield of dental anthropology aims to record, evaluate, and interpret both metric and nonmetric morphological traits of tooth crowns and roots. The initial description of tooth morphological traits was made by A. Hrdlicka in 1920 after observing characteristic shovel-shaped incisors.^16^ Various authors have emphasized that genetic factors such as homeobox (HOX) genes, mesenchymal regulatory molecules, and their receptors control tooth size and morphology.^17^

In our study, we recorded the prevalence of shoveling in the anterior teeth, examining their sexual dimorphism and occurrence in both the maxillary and mandibular arches and on the right and left sides.

## 13 Prevalence of shoveling and double shoveling

In our study, frequency of shoveling of incisors was 95.5 %, indicating that nearly all individuals exhibited this characteristic. The prevalence of shoveling in our study is significantly higher when compared to other studies. Sureshbabu et al. found the prevalence of shoveling to be 8.2 % in their study.^18^ In a study of the Bangalore population, which is racially mixed, the shoveling trait was significantly high in the Iranian group (80–84 %), followed by Christians, with the least expression in the Hindu group. This study used dental casts for recording.^19^ Another study conducted through a clinical examination on 6-10-year-old school children in an Indian population found the prevalence of shoveling to be 65.7 %.^20^ Similarly, Pillai et al. reported shoveling in 64 % of the overall sample of maxillary central incisors.^21^

In the Kerala population, the prevalence of shoveling was reported to be 69.12 %.^30^ However, in the Tamil population in Southern India, the shoveling trait was observed in only 8 % of the population.^22^ Kharat et al. found the incidence of shoveling among various populations to be: Syrians (5 %), Jordanians (5 %), Filipinos (6 %), Palestinians (7 %), Saudi Arabians (9 %), Pakistanis (9 %), Indians (12 %), Sudanese (20 %), Yemenis (20 %), and Egyptians (24 %).^12^ Similarly, Saini et al. reported a prevalence of 8.93 % for shovel-shaped incisors in the Riyadh population.^23^

In contrast, the Chinese population showed a high prevalence of shoveling at 80.4 %, observed on plaster casts, highlighting distinctive features of southern Chinese dentition.^24^ Conversely, a study on the Sri Lankan aboriginal Vedda population found that shoveling was absent (0 %), suggesting close ancestral ties to early South Asian populations.^25^ The prevalence of shoveling was observed to be comparatively lower in the South Indian population, with 81 % of the East Indian and 85 % of the West Indian population exhibiting this trait.^26^ Uthaman et al. reported a prevalence of 6.7 % among the Malayalee population in Coorg, in contrast to the Tibetan population, which showed a prevalence of 40 %.^27^ Sha et al. observed that shoveling was present in 65.2 % of the Tibeto-Nepalese population and 30.71 % of the Indo-Nepalese population.^13^ Nair et al. found a prevalence of 15 % for shoveling in the Kerala and Odisha populations.^28^ Additionally, a study conducted on the National Capital Region (NCR) observed a prevalence of 16.5 % for shoveling. In the Finnish population, the prevalence of shoveling was found to be 37.62 %.^29^

Double shoveling in our study was found to be present in 1.0 % of the Mysuru population. This finding contrasts with a study conducted among 6-10-year-old school children in the Bangalore population, which reported a prevalence of 66.6 %.^20^ Additionally, a study on Kerala population observed a prevalence of 12.64 %.^16^ Conversely, in the Sri Lankan aboriginal Vedda population, double shoveling was reported to be absent.^25^

These variations in prevalence rates highlight the significant regional differences in the expression of double shoveling traits, suggesting that genetic, environmental, and methodological factors may contribute to the observed discrepancies.

## 14 Sexual dimorphism of shoveling and double shoveling

It is well known that most of the nonmetric traits included in the ASUDAS exhibit low or no sexual dimorphism.^19^

In our study shoveling was found to be more prevalent in females than in males. In a study conducted on the Tamil Nadu population, it was observed that the prevalence of shoveling in males 50 (10 %) is slightly higher than in females 30(6 %).^22^ Conversely, a study conducted by Saini et al., on the Riyadh population observed that the prevalence of shoveling is higher in males 53(10.29 %) than in females 52(10.94 %).^23^ In a study conducted on the Brazilian population, it was observed that the prevalence of shoveling was more in males (15.5 %) than in females (13.2 %), with a p-value of 0.7152. Similarly, among the Chinese population, males exhibited a notably higher prevalence (81.6 %) than females (79.0 %).^24^

In the investigation among Finland's population, males exhibited a higher prevalence of shoveling (38.42 %) compared to females (36.62 %).^29^ Conversely, research by Sah et al. among Indo-Nepalese and Tibeto-Nepalese ethnic groups in the Western Hilly Region showed a contrasting pattern, with females demonstrating a higher prevalence (51.72 %) than males (39.53 %).^13^ These findings underscore the complexity of genetic and environmental influences on dental traits, contributing valuable insights to our understanding of gender-specific variations in dental morphology.

The study revealed a higher prevalence of double shoveling among females compared to males. This finding aligns with the results of a study conducted by Kapoor et al., which found that females exhibited a prevalence of 25.8 % for double shoveling, and males 18.5 %.^30^ Similarly, a study conducted on the NCR population indicated a higher prevalence of double shoveling in females (36 %) compared to males (22 %).^17^ These consistent observations across different populations suggest a potential gender-related variation in the expression of double shoveling, highlighting the need for further research into the underlying genetic and environmental factors that contribute to these differences.

## 15 Arch dimorphism of shoveling

In our study, shoveling exhibited a higher frequency in the upper arch (97.1 %) compared to the lower arch (2.9 %), with a highly significant p-value of 0.000, which is in consistent with several previous studies. Kharat et al. conducted a study on shovel-shaped incisors in Saudi Arabia, reporting a prevalence of 8.93 % in the upper arch.^23^ Similarly, among the Vedda population, shoveling was observed to be17.7 % in the upper arch.^25^ In the Kerala and Odisha populations studied by Nair et al., the frequency of shoveling in the upper arch was notably higher, with 84 cases observed in Kerala and 193 in Odisha. This pattern was further supported in the study by Baby et al., on Kerala population, where shoveling was more prevalent in the upper arch (51.8 %) compared to the lower arch (19.0 %).^16^ Contrastingly, study by Edgar et al., found a higher prevalence of shoveling in the lower arch (47.4 %) than in the upper arch (38.5 %).^31^ This discrepancy highlights potential regional variations or population-specific characteristics influencing the distribution of shoveling across dental arches.

Our study supports previous findings that shoveling is more common in the upper arch compared to the lower arch, which is consistent with observations in different populations. However, differences highlighted by Edgar et al. emphasize the complexity of shoveling's occurrence and indicate the necessity for additional regional studies to thoroughly understand these variations.

## 16 Quadrant dimorphism of shoveling

Our study observed variations in the prevalence of shoveling between the right and left central incisors. The trait was nearly equally distributed, with a prevalence of 50.3 % on the right side and 49.7 % on the left, indicating no significant difference. This finding aligns with some studies while differing from others. Hasegawa et al. reported a relatively low prevalence of shoveling in Mongolian females, with only 2.87 % observed on both the right and left sides.^11^ This finding contrasts with the study by Pillai et al., which demonstrated a higher prevalence of shoveling on the left side (64.0 %) compared to the right side (63.7 %) in the Gujarat population. However, the difference was not statistically significant, with a p-value of 0.257.^21^ Conversely, the Kerala study indicated that shoveling was slightly more prevalent on the right side (37.28 %) than on the left side (36.69 %).^16^ In a study of the Bangalore population, the prevalence of shovel-shaped incisors on right side (41 %) was higher than on the left side (40.25 %).^19^

Double shoveling exhibited an equal prevalence on both sides. This is consistent with findings from a Colombian study where the prevalence on the right side (13.57 %) was slightly lower than on the left side (14.11 %).^32^ Conversely, Baby et al. reported a higher prevalence of double shoveling on the right side (9.23 %) compared to the left side (7.14 %) in Kerala population.^16^ This indicates variability in the lateral distribution of double shoveling.

## 17 Class dimorphism of shoveling

Our study observed notable variations in the prevalence of shoveling among different upper and lower anterior teeth. The highest occurrence was found in the upper central incisors (92.2 %), followed by the upper lateral incisors 69.3 % and upper canines (20.1 %). In contrast, the lower anterior teeth exhibited significantly lower frequencies, with 2.8 % in the lower central incisors, 2.6 % in the lower lateral incisors, and 2.09 % in the lower canines. In the study by Sadhwani et al., Shoveling was present in 93 %, 88 %, 86 %, 72.9 % and 63.8 % of maxillary central incisors, maxillary lateral incisors, maxillary canines, mandibular lateral incisors, and mandibular central incisors respectively and shoveling was most commonly observed with maxillary canines.^31^ This pattern suggests that shoveling is predominantly an upper incisor trait, with minimal expression among lower incisors.

## 18 Grading in different class of teeth

Our study observed varying degrees of shoveling across different teeth, highlighting distinct grading patterns. Grade 1 was the most prevalent among all the tooth followed by grade 2 and grade 3. Higher grades were very rare, collectively making up for less than 7 %–2 %. The predominance of Grade 0 indicates minimal shoveling, which aligns with the overall lower prevalence of higher grades in the Mysuru population.

Similarly, a study by Chowdhry et al. on the NCR population, which only considered shoveling on the Lateral incisors (ULI-R and ULI-L), reported the following results: ULI-R showed Grade 0 in 20 %, Grade 1 in 29 %, Grade 2 in 34.5 %, Grade 3 in 14.5 %, and Grade 4 in 2 % of cases. ULI-L showed Grade 0 in 25 %, Grade 1 in 19.5 %, Grade 2 in 31.5 %, Grade 3 in 20.5 %, and Grade 4 in 3.5 % of cases.^17^

In a study conducted on the Gujarat population, the following prevalence was observed: UCI-R showed Grade 0 in 36.3 %, Grade 1 in 42.3 %, and Grade 2 in 21.4 % of cases. UCI-L showed Grade 0 in 36 %, Grade 1 in 42.7 %, and Grade 2 in 21.3 % of cases.^21^ In a study on the Vedda population, shoveling of Grade 0 in 82.3 %, Grade 1 in 9.8 %, and Grade 2 in 7.8 % was observed for upper central incisors (UI1).^25^ These comparisons highlight the variability in the prevalence and grading of shoveling traits across different populations. In the study by Sadhwani et al., most frequently observed grades were grade 0 with 31, grade 1 with 41 and 32, and grade 3 with 42.^31^

Our study observed varying degrees of double shoveling across different teeth, and was found to be predominantly absent in this population sample. Study by Chowdhry et al. on the NCR population, which only considered double shoveling on the left side (UCI-L), reported the following results: UCI-L showed Grade 0 in 50 %, Grade 1 in 21 %, Grade 2 in 15 %, Grade 3 in 4.5 %, Grade 4 in 8 % and Grade 5 in 1.5 % of cases.^17^ In a study on the Vedda population, Double shoveling was observed in upper central incisors (UI1) with Grade 0 in 100 % cases.^25^

## 19 Implementations in forensics

Analysis of nonmetric traits is a simple, easy, cost-effective method and requires minimal instruments and is reproducible. Our study highlights notable variations in the prevalence and distribution of the non-metric shoveling and double shoveling trait among Mysuru populations. The distinct prevalence patterns observed highlights the importance of regional studies. The results of this study can form the basic data for future studies. Studies have also shown a moderate or strong positive correlation between shoveling and other dental traits, dental anomalies and also between skeletal anomalies. Shoveling also expresses between generations indicating a strong genetic expression of the trait.^32^ These findings contribute valuable data for population affinity studies, finding ethnicity of the population regionally, aiding in ancestry determination, forensic identification and in understanding genetic and environmental influences on dental morphology.^31^

## 20 Strength and limitations of the study

Use of large sample size, use of standardized ASUDAS criteria, high intra-observer reliability and focus on an understudied population are the strength of our study.

The main limitation of our study is that it is hospital-based with random sampling. The sampling method may limit generalizability. Subjectivity in clinical scoring, despite high intra-observer reliability, Possible underestimation of trait prevalence due to exclusion of teeth with regressive changes and possibility of missing age-related variation in individuals over 40 years are the other limitations.

Our study used ASUDAS plaque as standard guidance for morphometric validation of the traits. However, no imaging techniques were used for radiographic validation in the study. In future studies, using methods like 3D scanning or micro-CT imaging could help obtain more accurate and measurable data of the different dental traits, allowing better comparison with other populations.

## 21 Conclusion

The present study provides significant insights into the prevalence and distribution of shoveling and double shoveling trait in the Mysuru population. The findings reveal that shoveling is, observed in the majority of individuals with no significant differences between genders and arch. However, the trait is markedly more frequent in the maxillary arch compared to the mandibular arch, reinforcing the anatomical variations commonly seen in different populations. However these findings cannot be generalizing beyond the studied population. More such regional studies with large sample and strong methodology would benefit the forensic community.

## Patient/guardian consent

Not Applicable.

## Support

Financial support by JSSAHER.

## Declaration of competing interest

The authors declare that they have no known competing financial interests or personal relationships that could have appeared to influence the work reported in this paper.
